# Persistence of Th17/Tc17 Cell Expression upon Smoking Cessation in Mice with Cigarette Smoke-Induced Emphysema

**DOI:** 10.1155/2013/350727

**Published:** 2013-12-29

**Authors:** Min-Chao Duan, Hai-Juan Tang, Xiao-Ning Zhong, Ying Huang

**Affiliations:** ^1^Department of Respiratory Medicine, The First Affiliated Hospital of Guangxi Medical University, Nanning, Guangxi 530021, China; ^2^Department of Respiratory Medicine, The Eighth People's Hospital of Nanning, Nanning, Guangxi 530001, China

## Abstract

Th17 and Tc17 cells may be involved in the pathogenesis of chronic obstructive pulmonary disease (COPD), a disease caused predominantly by cigarette smoking. Smoking cessation is the only intervention in the management of COPD. However, even after cessation, the airway inflammation may be present. In the current study, mice were exposed to room air or cigarette smoke for 24 weeks or 24 weeks followed by 12 weeks of cessation. Morphological changes were evaluated by mean linear intercepts (Lm) and destructive index (DI). The frequencies of CD8^+^IL-17^+^(Tc17) and CD4^+^IL-17^+^(Th17) cells, the mRNA levels of ROR gamma and IL-17, and the levels of IL-8, TNF-alpha, and IFN-gamma in lungs or bronchoalveolar lavage fluid of mice were assayed. Here we demonstrated that alveolar enlargement and destruction induced by cigarette smoke exposure were irreversible and that cigarette smokeenhanced these T-cell subsets, and related cytokines were not significantly reduced after smoking cessation. In addition, the frequencies of Th17 and Tc17 cells in lungs of smoke-exposed mice and cessation mice were positively correlated with emphysematous lesions. More important, the frequencies of Tc17 cells were much higher than Th17 cells, and there was a significantly positive correlation between Th17 and Tc17. These results suggested that Th17/Tc17 infiltration in lungs may play a critical role in sustaining lung inflammation in emphysema. Blocking the abnormally increased numbers of Tc17 and Th17 cells may be a reasonable therapeutic strategy for emphysema.

## 1. Introduction

Chronic obstructive pulmonary disease (COPD) is characterized by persistent airflow limitation due to airway obstruction and emphysematous destruction [1]. Smoking is the most important etiological factor in the development of airway inflammation in COPD [1]. Until now, smoking cessation is regarded as the most important intervention in reducing the progression of COPD [2]. However, in those who develop COPD this inflammatory response persists after smoking cessation, suggesting an abnormal regulation mechanism similar to those occurring in autoimmune disorders.

COPD shares some features with autoimmune diseases [3–5]. Th1/Tc1 cells contribute principally, but not exclusively, to the pathogenesis of COPD. Th17 cells are now defined as a separate CD4^+^ T-cell subset distinct from the Th1 and Th2 cells, with the expression of distinctive transcription factor ROR-ct (RAR-related orphan nuclear receptor ct in mice; RORC, RAR-related orphan nuclear receptor C in humans). IL-17A, IL-17F, IL-21, and IL-22 are secreted by and involved in the in vivo function of Th17 cells [6]. Th17 cells are the primary drivers of inflammatory responses in several T cell driven autoimmune diseases, such as rheumatoid arthritis and multiple sclerosis, which were previously thought to be exclusively mediated by Th1 cells [7, 8]. More recently, a completely new subset of CD8^+^ T cells producing IL-17 (Tc17) was also discovered. Similar to Th17 cells, Tc17 cells secrete IL-17A and IL-17F and are implicated in the pathogenesis of some human autoimmune diseases, such as psoriasis and systemic lupus erythematosus (SLE) [9, 10]. Our previous studies have demonstrated that increased Th17 and Tc17 cells were found in lungs of emphysema group and that the overrepresentation of these T-cell subsets might be due to their differentiation and expansion stimulated by local proinflammatory cytokines and to recruitment into lungs via CCR6/CCL20 [11, 12]. However, the role of Tc17 cells and the correlation between Tc17 cells and Th17 cells in COPD have not been systematically investigated. In addition, the effects of smoking cessation on pathological and inflammatory changes mediated by Th17 and Tc17 cells are also far less clear.

We hypothesized that Tc17 and Th17 cells are involved in the sustained airway inflammatory response and play a key role in the immunopathology of emphysema. To test this hypothesis, we evaluated the expressions of CD4^+^IL-17^+^ (Th17) cells and CD8^+^IL-17^+^ (Tc17) cells in lungs of mice exposed to cigarette smoke or room air for 24 weeks followed by 12 weeks of cessation and analyzed the correlation between Th17 and Tc17 cells in smoke-exposed mice, and their relationships with emphysematous lesions. Finally, we tested the concentrations of IL-8, TNF-*α*, and INF-*γ* in BALF and the mRNA expressions of IL-17 and ROR*γ*t in lungs.

## 2. Materials and Methods

### 2.1. Animals

Male BALB/c mice (8 weeks of age, 20–25 g body weight) were purchased from The Guangxi Medical University Laboratory Animal Center (Nanning, China). All mice were housed in sterilized cages and maintained on a 12:12-h light-dark cycle and received sterilized food and water ad libitum. The Ethics Committee for Animal Research of Guangxi Medical University approved all animal experiments.

### 2.2. Cigarette Smoke Exposure

Male BALB/c mice were divided into three groups (*n* = 20 per group). The first group (nonesmoke mice) used as control was exposed to room air for 24 weeks, the second group (smoke-exposed mice) was exposed to 5 cigarettes (Nanning Jiatianxia unfiltered cigarettes: 12 mg of tar and 0.9 mg of nicotine) four times per day with 30 minutes smoke-free intervals in a closed 0.75-m^3^ room, 5 days per week, for 24 weeks, and the third group (cessation mice) was exposed to cigarette smoke for 24 weeks and then housed unexposed for 12 weeks. Mice tolerated cigarette smoke exposure without evidence of toxicity (carboxyhemoglobin levels ~10% and no weight loss). An optimal smoke air ratio of 1 : 6 was obtained. All mice were housed in accordance with institutional guidelines. The mice were sacrificed 24 hours after the last air or smoke exposure, or after the smoke-free period of 12 weeks.

### 2.3. Sample Collection and Processing

Twenty-four hours after the last exposure, mice were weighed and sacrificed with an overdose of pentobarbital (Sanofi-Synthelabo). In some mice, the left lung was used for RNA analysis, and the right lung was used for preparation of single-cell suspensions. In other mice, the left lung was inflated by instilling 10% formalin at a constant pressure of 25 cm H_2_O (for 10 min) and then ligated and removed. Inflated lungs were fixed for 24 h before embedding in paraffin. Serial midsagittal sections were obtained for morphological and histological analyses. The right lung was lavaged 6 times through a tracheal cannula with 0.75 mL saline (NaCl 0.9%), prewarmed at 37°C. More than 90% of BALF was collected from each animal and was centrifuged at 400 g for 5 minutes at 4°C to remove cell debris. The supernatants were used for ELISA analysis.

### 2.4. Histology and Morphometric Analysis

After fixation, midsagittal sections were stained with hematoxylin and eosin for histological analysis. For each animal, 10 fields at a magnification of 100x were captured randomly from the 4 different zones of the left lung. We determined enlargement of alveolar spaces by quantifying the mean linear intercept (Lm) and destruction of alveolar walls by measuring the destructive index (DI) in all mice, as described previously [13, 14]. Two investigators independently measured Lm and DI in a blinded manner.

### 2.5. Preparation of Lung Single-Cell Suspensions

Lung single-cell suspensions were prepared from the right lung, as detailed previously [15]. Briefly, the lung was thoroughly minced, digested, passed through a 70-*μ*m cell strainer, washed twice with cold PBS at 300 ×g for 10 min at 4°C, and resuspended in PBS. The mononuclear cells were isolated by Ficoll-Plaque (Solarbio Science & Technology, China) gradient centrifugation from the lung single-cell suspensions, washed twice with PBS, and kept on ice until labelling.

### 2.6. Immunofluorescence Labeling and Flow Cytometry

The expression markers on T cells were determined by flow cytometry after surface staining or intracellular staining with anti-mouse-specific Abs conjugated with PE-Cy5, FITC, and PE. These mouse Abs included anti-CD4, anti-CD8, and anti-IL-17 mAbs, which were purchased from BD Biosciences or eBioscience (San Diego, CA). Cell surface stainings were performed according to standard procedures using mAbs against CD4 and CD8 directly conjugated to PE-Cy5 and FITC prior to cell permeabilization. Intracellular staining for IL-17 was performed with PE-labeled mAbs. Briefly, Cells were incubated for 5 h at 37°C in 5% CO_2_ in the presence of PMA (25 ng/mL, Sigma-Aldrich, USA), ionomycin (10 *μ*g/mL, Sigma-Aldrich, USA), and GolgiStop (BD Biosciences). After incubation, the cells were stained with fluorescent antibodies against CD4 and CD8 at room temperature in the dark. After surface staining, the intracellular IL-17 was then stained with fluorescent antibodies against IL-17 using the eBioscience fixation/permeabilization and permeabilization buffers according to the manufacturer's recommended protocol [16]. Isotype controls were given to enable correct compensation and confirm antibody specificity. Flow cytometry was performed on a BD FACS Calibur flow cytometer and analyzed using FCS Express V4 software.

### 2.7. Real-Time Quantitative PCR

Total RNA was extracted from lung tissue with TRIzol **(**Invitrogen-Life Technologies) according to the manufacturer's instructions. cDNA was prepared using oligo(dT) primers (RevertAid First Strand cDNA Synthesis Kit, Fermenta). Quantitative RT PCR was performed by duplicate with SYBR Green I using a LightCycler (iCycler IQ, BioRad, American) according to the manufacturer's instructions. The PCR conditions were (94°C for 20 s 57.5°C for 30 s 72°C for 31 s) × 40 cycles for *β*-actin expression, (95°C for 5 s 60°C for 34 s) × 35 cycles for IL-17A expression, and (94°C for 20 s 58°C for 30 s 72°C for 31 s) × 40 cycles for ROR*γ*t expression. The following primers were used: 5′-ATCCACGAAACTACCTTCAA-3′ and 5′-ATCCACACGGAGTACTTGC-3′ for *β*-actin; 5′-GGAAAGCTGGACCACCACA-3′ and 5′-CACACCCACCAGCATCTTCTC-3′ for IL-17A; and 5′-ACGGCCCTGGTTCTCATCA-3′ and 5′-CCAAATTGTATTGCAGATGTTCCAC-3′ for ROR*γ*t. The band sizes of the fragments were 117 bp (IL-17A), 79 bp (ROR*γ*t), and 200 bp (*β*-actin). The identity of the amplified products was examined using 12% polyacrylamide gel electrophoresis and melt curve analysis, and the ratios of each gene product to *β*-actin product were used as indices of IL-17 mRNA and ROR*γ*t mRNA expression.

### 2.8. Cytokine Measurement

The concentrations of IL-8, TNF-*α*, and INF-*γ* in BALF were measured by ELISA kits according to the manufacturer's protocols (all kits were purchased from R&D Systems, Minneapolis, MN). All samples were assayed in duplicate.

### 2.9. Statistical Analysis

Quantitative data were expressed as the mean ± SEM. Differences between the groups were analysed using an analysis of variance (ANOVA). When the significant differences were detected, the ANOVA was followed by an unpaired two-tailed Student's *t-*test. Correlation coefficients were calculated using Pearson's rank correlation test. A *P* value < 0.05 was considered statistically significant. All statistical analyses were performed by using SPSS statistical software version 16 (SPSS Inc., Chicago, IL).

## 3. Results

### 3.1. Alveolar Enlargement and Destruction Induced by Cigarette Smoke Exposure Are Irreversible

**Figure 1:**
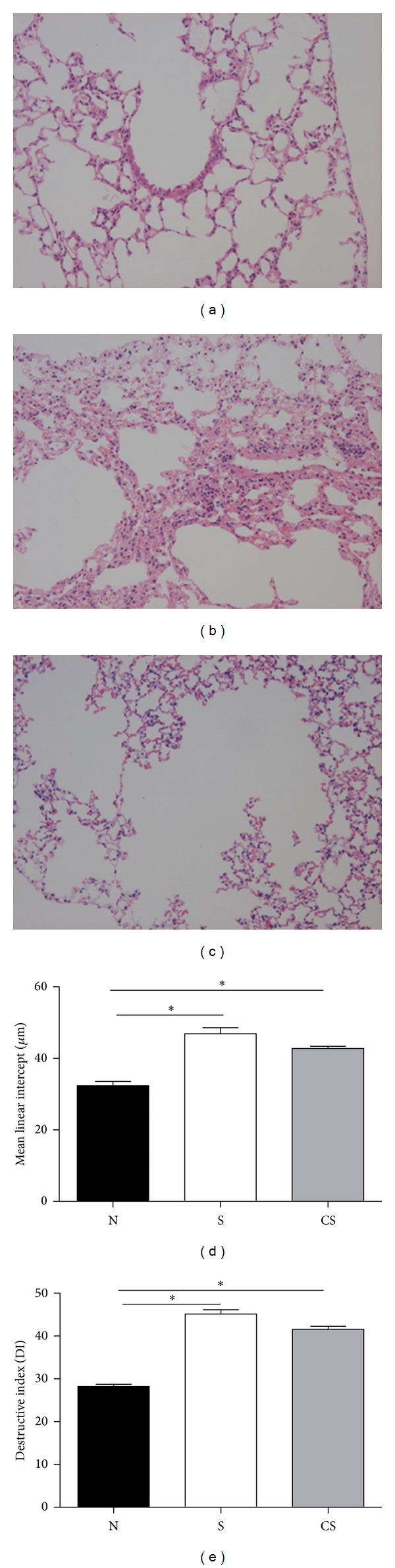
Cigarette smoke-induced alveolar enlargement is irreversible. Representative photomicrographs of hematoxylin and eosin stained lung tissue of nonsmoke mice (a), smoke-exposed mice (b), and smoking cessation mice (c). Magnification ×100. Morphometry of the lungs in mice exposed to air, mice exposed to cigarette smoke for 24 weeks, and mice exposed to cigarette smoke for 24 weeks plus a smoking cessation period of 12 weeks: (d) Lm and (e) DI values of mice. Results are expressed as means ± SEM. *n* = 10 animals/group; *P < 0.05.

Emphysema is a structural disorder characterized by destruction of the alveolar walls and enlargement of the alveolar spaces. Histologically, the lung sections from the air-exposed mice showed normal alveolar structure (Figure 1(a)). In contrast, the lung sections from the smoke-exposed mice showed enlargement of the air spaces accompanied by the destruction of the normal alveolar architecture (Figure 1(b)). The alveolar enlargement and destruction are still present after a smoking cessation period of 12 weeks (Figure 1(c)).

The results of the morphometric investigation of the lungs are given in Figures 1(d) and 1(e). We determined enlargement of alveolar spaces by quantifying the mean linear intercept (Lm) and destruction of alveolar walls by measuring the destructive index (DI). Compared with nonsmoke mice, Lm was significantly increased in smoke-exposed mice and smoke cessation mice, and there was no significant difference in smoke-exposed mice compared to smoke cessation mice (Figure 1(d)). DI was also significantly higher in smoke-exposed mice and cessation mice than in nonsmoke mice, and there was no difference between cessation mice and smoke-exposed mice (Figure 1(e)).

### 3.2. Elevated Levels of Interleukin (IL)-8 and TNF-*α* in BALF Are Irreversible after Smoking Cessation

**Figure 2:**
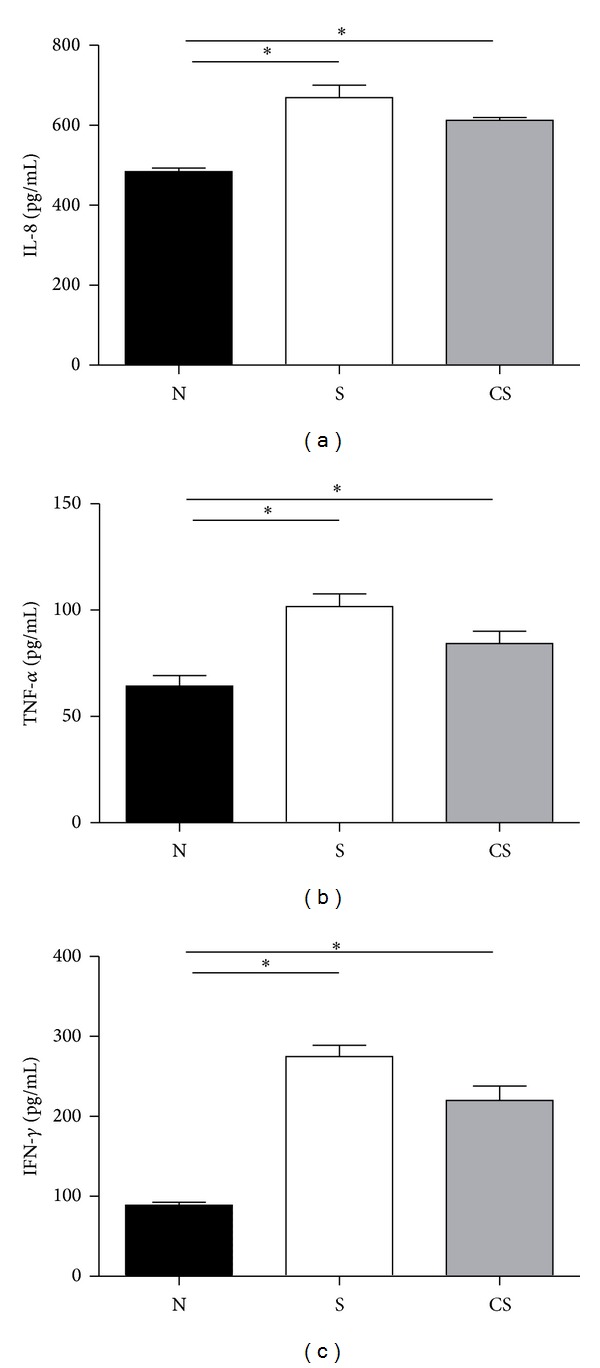
Elevated levels of interleukin (IL)-8, TNF-*α*, and IFN-*γ* are not reversible after smoking cessation. Protein levels of IL-8, TNF-*α*, and IFN-*γ* in BALF of nonsmoke mice, smoke-exposed mice, and smoke cessation mice were measured by using ELISA. (a) Protein levels of IL-8 in BALF, (b) protein levels of TNF-*α* in BALF, (c) protein levels of IFN-*γ* in BALF. Results are expressed as pg/mL (mean ± SEM). *n* = 10 animals/group; *P < 0.001.

Chronic inflammation in the airways contributes, in a causal way, to the genesis of alveolar enlargement [17]. Thus, we measured the levels of IL-8, TNF-*α*, and IFN-*γ* in BALF of mice. Results showed that the levels of IL-8, TNF-*α*, and IFN-*γ* in BALF of smoke cessation mice and smoke-exposed mice were markedly higher than those of normal controls, and there was no significant difference between cessation mice and smoke-exposed mice (Figures 2(a), 2(b), and 2(c)). These data suggested that proinflammatory activity might not change after smoking cessation.

### 3.3. Increased Numbers of CD4^+^IL-17^+^ (Th17) Cells and CD8^+^IL-17^+^ T (Tc17) Cells Are Irreversible after Smoking Cessation

**Figure 3:**
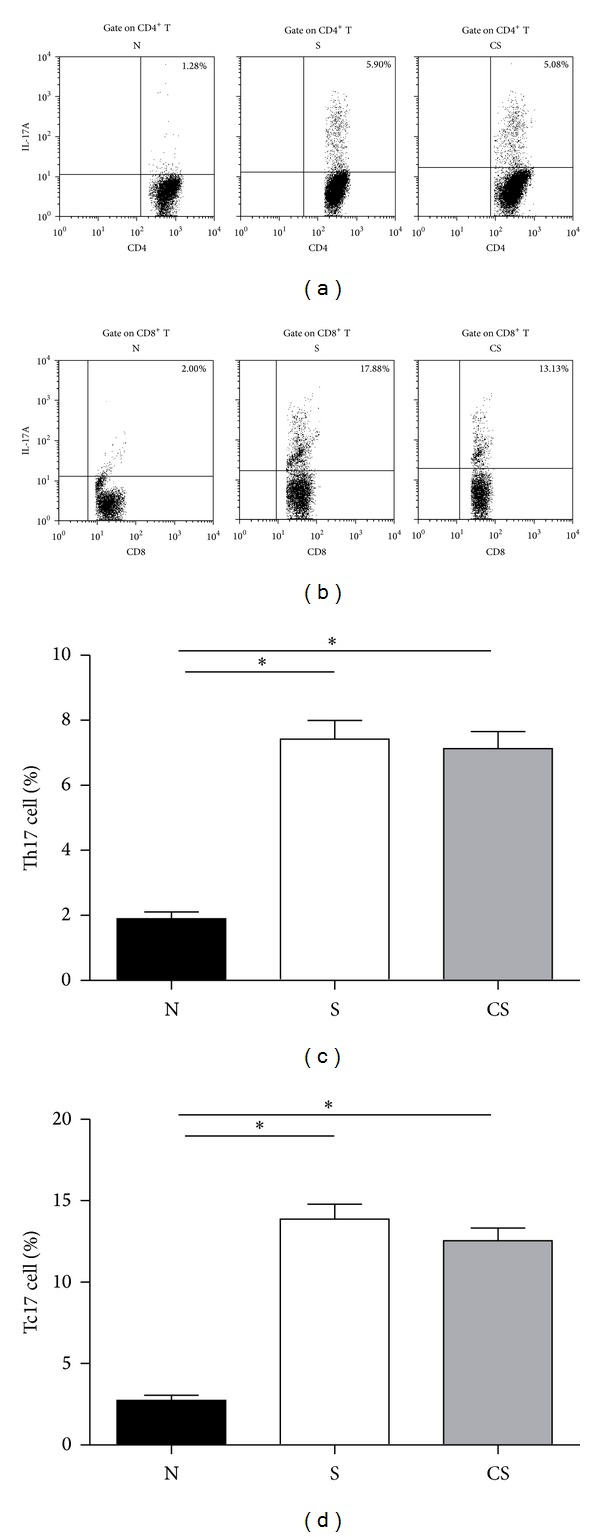
Increased numbers of CD4^+^IL-17^+^ (Th17) cells and CD8^+^IL-17^+^ T (Tc17) cells are not reversible after smoking cessation.Lung single-cell from nonsmoke mice, smoke-exposed mice, and smoke cessation mice was stimulated by PMA and ionomycin for 4 h and analyzed IL-17A by using flow cytometry. (a) Representative FACS staining for IL-17A in gated CD4^+^ T cells. (b) Representative FACS staining for IL-17A in gated CD8^+^ T cells. (c) The frequencies (%) of Th17 cells; (d) the frequencies (%) of Tc17 cells. Results are expressed as % (mean ± SEM). *n* = 10 animals/group; *P < 0.05.

To address the role of Th17 and Tc17 cells in the pathogenesis of CS-induced pulmonary responses, we used flow cytometry to identify the expression of these T-cell subsets in lungs of nonsmoke mice and smoke-exposed mice before and after smoking cessation. Lung single cells were labeled with different surface markers. The following subsets of CD4^+^IL-17^+^ (Th17) cells and CD8^+^IL-17^+^ T (Tc17) cells were detected following phorbol 12-myristate 13-acetate (PMA) and ionomycin stimulation. Representative flow cytometry results were shown in Figures 3(a) and 3(b), and the quantitative results were shown in Figures 3(c) and 3(d). Compared with nonsmoke mice, the frequencies of Th17 cells were significantly increased in smoke-exposed mice and smoke cessation mice, and there was no significant difference in smoke-exposed mice compared to cessation mice (Figure 3(c)). The frequencies of Tc17 cells were also significantly higher in smoke-exposed mice and cessation mice than in nonsmoke mice, with no significant difference between cessation mice and smoke-exposed mice (Figure 3(d)). In addition, we noted that the frequencies of Tc17 cells were much higher than Th17 cells in lungs of smoke-exposed mice and smoking cessation mice.

### 3.4. Elevated mRNA Expressions of ROR*γ*t and IL-17 Are Irreversible after Smoking Cessation

**Figure 4:**
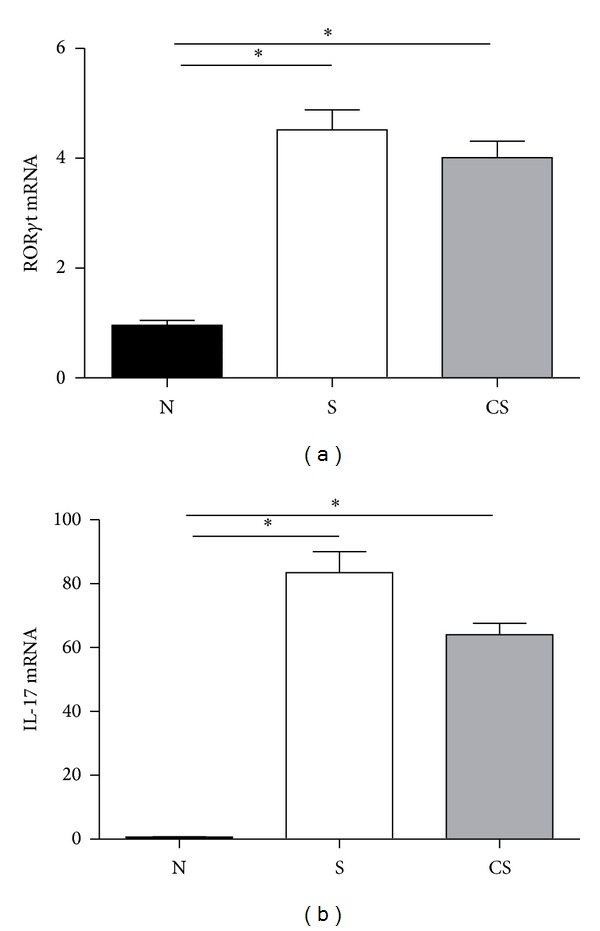
Elevated mRNA expressions of ROR*γ*t and IL-17 are not reversible after smoking cessation. The mRNA expressions of ROR*γ*t and IL-17 in lungs of nonsmoke mice, smoke-exposed mice, and smoke cessation mice were measured by using ELISA. (a) mRNA expressions of ROR*γ*t in lungs; (b) mRNA expressions of IL-17 in lungs. Results are expressed as mean ± SEM. *n* = 10 animals/group; *P < 0.001.

ROR*γ*t was described as an important transcription factor involved in the development and function of Th17 and Tc17 cells and appears to be the most specific molecular marker available to date. Th17 and Tc17 cells can produce IL-17A and IL-17F [6, 18]. Since chronic cigarette smoke-exposure induces increased proportions of Th17 and Tc17 cells in the lungs, we investigated the mRNA expression levels of ROR*γ*t and IL-17 by real-time QPCR. Compared with nonsmoke mice, the ROR*γ*t mRNA levels were significantly increased in smoke-exposed mice and smoke cessation mice, and there was no significant difference in smoke-exposed mice compared to cessation mice (Figure 4(a)). Similarly, compared with nonsmoke mice, the IL-17A mRNA levels were also markedly higher in smoke-exposed mice and cessation mice, with no significant difference between the latter groups (Figure 4(b)).

### 3.5. Correlation between Different Parameters

**Figure 5:**
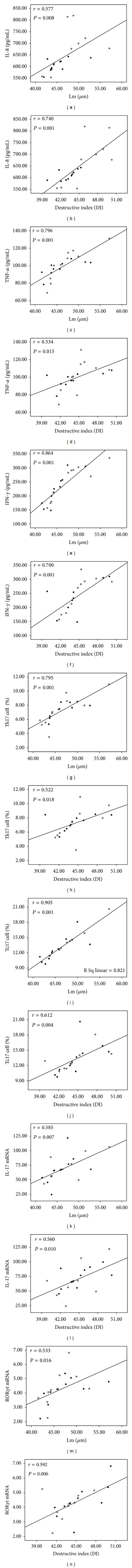
Correlations between (a) the protein levels of IL-8 in BALF and Lm, (b) the protein levels of IL-8 in BALF and DI, (c) the protein levels of TNF-*α* in BALF and Lm, (d) the protein levels of TNF-*α* in BALF and DI, (e) the protein levels of IFN-*γ* in BALF and Lm, (f) the protein levels of IFN-*γ* in BALF and DI, (g) the frequency of CD4^+^IL-17^+^ Th17 cells in lungs and Lm, (h) the frequency of CD4^+^IL-17^+^ Th17 cells in lungs and DI, (i) the frequency of CD8^+^IL-17^+^ Tc17 cells in lungs and Lm, (j) the frequency of CD8^+^IL-17^+^ Tc17 cells in lungs and DI, (k) the levels of IL-17 mRNA in lungs and Lm, (l) the levels of IL-17 mRNA in lungs and DI, (m) the levels of ROR*γ*t mRNA in lungs and Lm, and (n) the levels of ROR*γ*t mRNA in lungs and DI. Data were determined by Pearson's rank correlation coeficients. Smoke-exposed mice (open circle) smoke cessation mice (closed circles).

To further confirm our results, the smoke-exposed mice and the smoke cessation mice were grouped together, and the relationship between the various parameters was calculated. In BALF, the levels of IL-8, TNF-*α*, and INF-*γ* were positively correlated with Lm (*r* = 0.577, *P* < 0.01, *r* = 0.577, *P* < 0.01 and *r* = 0.796, *P* < 0.001, resp.) (Figures 5(a), 5(b), and 5(c)) and with DI (*r* = 0.74, *P* < 0.001, *r* = 0.577, *P* < 0.01 and *r* = 0.534, *P* < 0.01, resp.) (Figures 5(d), 5(e), and 5(f)). In lungs, the frequencies of Th17 and Tc17 cells were positively correlated with Lm (*r* = 0.795, *P* < 0.001 and *r* = 0.906, *P* < 0.001, resp.) (Figures 5(g) and 5(h)) and with DI (*r* = 0.522, *P* < 0.001 and *r* = 0.612, *P* < 0.01, resp.) (Figures 5(i) and 5(j)). Similarly, the levels of IL-17 and ROR*γ*t mRNA were also positively correlated with Lm (*r* = 0.560, *P* < 0.01 and *r* = 0.533, *P* < 0.05, resp.) (Figures 5(i), 5(k), and 5(l)) and with DI (*r* = 0.585, *P* < 0.005 and *r* = 0.592, *P* < 0.01, resp.) (Figures 5(m) and 5(n)). In addition, the frequencies of Th17 and Tc17 cells revealed strong correlation with the IL-17 mRNA (data not shown) and with the ROR*γ*t mRNA (data not shown). Furthermore, a highly significant correlation between the frequencies of Tc17 cells and the frequencies of Th17 cells was also found (data not shown).

## 4. Discussions

The objective of this study was to investigate the effects of cigarette smoke exposures on Th17/Tc17 cells and related proinflammatory cytokines and to assess the reversibility of these effects following smoking cessation. In this study, we demonstrated that chronic cigarette smoke exposure results in alveolar enlargement and destruction and increases the numbers of Th17/Tc17 cells, the levels of IL-17 and ROR*γ*t mRNA, and the protein levels of IL-8, TNF-*α* and INF-*γ* in lungs or BALF. We also demonstrated that alveolar enlargement and destruction induced by cigarette smoke exposure were irreversible and that cigarette smokeenhanced these T-cell subsets, and cytokines were not significantly reduced after smoking cessation. Lastly, the frequencies of Th17 and Tc17 cells in lungs of smoke-exposed mice and cessation mice were positively correlated with emphysematous lesions. Interestingly, the frequencies of Tc17 cells were much higher than Th17 cells, and Tc17 cells were positively correlated with Th17 cells. These results suggested that Th17 and Tc17 cells infiltrations in lungs may play a critical role in sustaining lung inflammation in emphysema.

In this present study, airspace enlargement in murine model of lung emphysema was evident after 24 weeks of cigarette smoke exposure. The enlargement and destruction were not significantly reduced after smoking cessation, suggesting that induction of lung emphysema by alveolar wall destruction is not reversible. These findings are in agreement with the in vivo data of Braber et al. [19], who demonstrated that emphysema was still present in mice after smoke exposure followed by a smoking cessation period. Additionally, the persistent emphysema observed in murine model is also similar to findings in people who have stopped smoking [20–22].

Cigarette smoke can act on airway epithelial cells and alveolar macrophages to release several inflammatory mediators, such as IL-1, IL-8, TNF-*α*, and INF-*γ* [23]. It has been demonstrated that the inflammatory response in cigarette smoke-induced pulmonary damage is characterized by an increased number of Th1/Tc1 cells and that IFN-*γ* is a potent activator of the extrinsic/death receptor and intrinsic/mitochondrial apoptosis pathways and leads to lung inflammation and emphysema associated with induction of matrix metalloproteinase 12 [24]. IL-8 may activate and recruit neutrophils to mediate the inflammatory response in COPD [25]. TNF-*α* can also stimulate epithelial cells to release monocytes/macrophage- or neutrophil-derived chemotactic factors [26], leading to injury and remodeling of lung tissue. However, little is known about these cytokines levels in the BALF after smoking cessation. In the current study, we found that the IL-8, TNF-*α*, and IFN-*γ* levels in BALF were significantly higher in smoke-exposed mice and cessation mice than in nonsmoke mice, and there was no significant difference between smoke-exposed mice and cessation mice. Additionally, BALF IL-8, TNF-*α*, and IFN-*γ* levels positively correlated with emphysematous lesions, respectively. These findings indicate that proinflammatory activity changes in the airways of smoke-exposed mice are irreversible after smoking cessation and that the persistent airway inflammation induces ongoing lung tissue damage. Further investigations are needed and should be considered important as it could in part explain the reasons of why some exsmokers develop COPD and some do not.

Recent studies have demonstrated that Th17 cells were increased in tissues from patients with emphysema, and models of this disorder [27–29] have led to the speculation that Th17 cell response plays a critical role in the pathogenesis of emphysematous tissue destruction. The Tc17 subset is a new unique cell lineage, which displays a greatly suppressed cytotoxic function and shares some key features with the Th17 subset [30]. More recently, it has been shown that CD8 positive T-cells expressed IL-17 A and IL-17F were increased in the airways of COPD patients, which indicates that this subset plays a significant role in the pathogenesis of COPD [31]. In accord with above results, our previous data have shown that the numbers of Th17 and Tc17 cells were significantly elevated in lungs of smoke-exposed mice compared to air-exposed mice and that Th17/Tc17-driven immune responses were possibly associated with emphysema. However, little is known about the Th17/Tc17 cell changes in emphysema after smoking cessation. Our present works have extended the previous studies and further demonstrated that the increased numbers of Th17 and Tc17 cells were found in the lungs of cigarette smoke-exposed mice. After smoking cessation, the numbers of Th17 and Tc17 cells were still significantly increased compared to the air-exposed animals. Of interest, our data showed that, compared to control animals, the percentages of Tc17 cells in lungs are generally higher than those of Th17 cells. These data were in agreement with a previous report by Maeno et al. [32], who found that pulmonary tissue T-cell infiltration associated with chronic smoke exposure consisted for the most part of CD8^+^ cytotoxic T cells. Thus, we presume that the Tc17 subset might play a more important role in the elicitation of COPD. In addition, the positive correlation between Th17 and Tc17 cells was found in smoke-exposed mice and smoking cessation mice. Furthermore, the Tc17 differentiation is similar to the one described for Th17 cells [30]. Taken together, these data suggest that activated Th17 and Tc17 subsets either preferential homing to the lungs or proliferate at these sites after cigarette smoke exposure and that Tc17 cells may cooperate with Th17 cells in similar functions in ongoing airway inflammation in emphysema. However, the mechanisms underlying the correlation between Tc17 and Th17 should be clarified in future studies.

ROR*γ*t and RORc can induce Th(c)17 cell differentiation in mice or in human [33, 34]. ROR*γ*t directly binds to the specific ROR responsive element in the IL-17 gene promoter region, leading to increased IL-17 expression [35]. IL-17 can induce pulmonary inflammation and tissue destruction, specifically through upregulation of MMP9 and its effects as a neutrophil chemoattractant [36]. IL-17 can also stimulate mucin production by respiratory epithelial cells [37]. In the present study, significantly elevated levels of IL-17 and ROR*γ*t mRNA were found in lungs from the smoke-exposed mice. In addition, a positive correlation between levels of IL-17 and ROR*γ*t mRNA and emphysematous lesions was found. These observations are consistent with a number of recent reports demonstrating that an enhanced expression of IL-17 and ROR*γ*t was also observed in the pulmonary vessels and arteries of COPD patients [33]. Furthermore, we noticed a strong positive correlation between the frequency of Th17 and Tc17 cells and the mRNA expression of IL-17 and ROR*γ*t. Importantly, the increased IL-17 and ROR*γ*t mRNA induced by cigarette smoke were irreversible after smoking cessation. Taken together, these studies indicated that, upon stimulation with cigarette smoking, Tc17 and Th17 cells might differentiate and develop under the induction of ROR*γ*t and IL-17 and then recruit and infiltrate neutrophils to the sites to damage lung tissues. After smoking cessation, these activated cells may modulate ongoing inflammatory in the lungs.

In conclusion, chronic cigarette smoke exposure may lead to irreversible lung damage. After the typical pathologic changes took place, the increased BALF IL-8, TNF-*α*, and IFN-*γ* levels, lungs Th17/Tc17 frequencies, and IL-17 and ROR*γ*t mRNA could hardly be reversible. Additionally, the frequencies of Tc17 cells were much higher than Th17 cells in lungs of smoke-exposed mice and cessation mice. More importantly, the frequencies of lung Th17/Tc17 cells, the expressions of lung IL-17 and ROR*γ*t mRNA, and the levels of BALF IL-8,  TNF-*α*, and IFN-*γ* from all smoke-exposed mice were positively correlated with emphysematous lesions. And a strong positive correlation between Tc17 cells and Th17 cells was also observed. These studies suggest that the presence of Th17 and Tc17 cells might contribute to the pathogenesis of CS-induced persistent inflammation and alveolar destruction. Although the Th17/Tc17 cell numbers and the cytokines and transcription factor levels do not significantly decrease up to 12 weeks after smoking cessation, we cannot exclude the possibility that a decrease could take place the following weeks. Thus smoking cessation should be the first step in reducing the progression of lung emphysema, and blocking Th17/Tc17 cells, and related cytokines may be additional possible therapeutic approach for emphysema.
